# Bi‐phasic Microtubule Defects in Tauopathy: Treatment Implications

**DOI:** 10.1002/alz.089658

**Published:** 2025-01-03

**Authors:** Xiaohuan Sun, Peter W Baas, Celeste M. Karch, Liang Qiang

**Affiliations:** ^1^ Drexel University college of medicine, Philadelphia, PA USA; ^2^ The Charles F. and Joanne Knight Alzheimer Disease Research Center, Washington University, St. Louis, MO USA

## Abstract

**Background:**

In tauopathies, such as Frontotemporal Dementia (FTD), tau loses association with microtubules (MTs) and forms neurofibrillary tangles. Tau is an abundant MT‐associated protein in neurons, which essentially regulate MT properties. Because pathological tau binds less avidly to MTs, which was thought to reduce the levels and stability of axonal MTs. However, this idea is based on the dogma of functional tau as a MT stabilizer, which was challenged by some recent studies via tau‐knockdown strategies. The situation is further confounded in tauopathies because the pathological tau may elicit certain gain‐of‐toxicities on axonal MTs.

**Method:**

To investigate this matter in a more disease‐relevant scenario, we used 1) cerebral organoids generated from an FTD‐patient derived induced pluripotent stem cells bearing a tau mutation (P301S/P301L/R406W) and their isogenic control corrected by CRISPR‐Cas9; and 2) postmortem brain tissues from a cohort of FTD patients, as our models.

**Result:**

Elevated soluble tau was identified in tau^P301S^ organoids during the early development with an increased ratio of mature/immature isoforms (4R‐tau/3R‐tau); whereas an overt reduction in soluble tau was identified in late‐stage developed organoids and postmortem FTD brains accompanied by increased aberrant PTM‐modifications of tau. Strikingly, MAP6, a bona fide MT stabilizer that competes with tau’s effects on MTs, presented opposite changes in its levels in tau^P301S/R406W^ organoids at the early‐ and late‐developed stages also in the postmortem brain model as the late‐developed stage of disease. Such bi‐phasic changes in tau and MAP6 were consistently reflected by the predicted changes in MT dynamics. Interestingly, certain neurodegenerative features recapitulated in tau^P301S^ organoids were ameliorated by novel corrective strategies such as tau antisense oligonucleotides in the early stage of disease.

**Conclusion:**

We conclude that the biphasic changes in tau pathology with the subsequent MT defects may underlie the progressive neurodegeneration in tauopathy. Thus, therapies to correct tau and MT abnormalities in tauopathy must be vetted for the specific stages of the disease

